# Should Industry Speak the Loudest in Informing the Public on Medical Matters?

**DOI:** 10.1371/journal.pmed.0030118

**Published:** 2006-02-28

**Authors:** Gordon McCarter

**Affiliations:** **1**Touro UniversityVallejo, CaliforniaUnited States of America

Lacasse and Leo make an important contribution in the debate over prescription drug advertising [ 1]. Their demonstration of the ubiquity of the scientifically unsupported claim that serotonin reuptake inhibitors (SSRIs) “correct a chemical imbalance” points out the need for better regulation of direct-to-consumer (DTC) advertising by the United States Food and Drug Administration (FDA). This question then leads to a larger issue: how does the public get unbiased information to rationally choose between different treatment options that may be available for particular diseases.

First, it should be emphasized that the “monoamine hypothesis” of depression has not been proven wrong; it is simply very incomplete. Inhibiting the serotonin and/or norepinephrine transporter(s) clearly relieves the symptoms of major depression for many patients, and the safety profiles of the current SSRIs and serotonin/norepinephrine reuptake inhibitors (SNRIs) are quite favorable. Nevertheless, the exclusively pharmaceutical and commercial approach to the treatment of depression that the typical consumer is exposed to in the media does a grave disservice to those who might benefit from the many other treatment options available.

The realization that depression is a true physical disease has allowed millions of sufferers to seek treatment, and has removed much of the guilt and shame (or proud forbearance) that sometimes resulted from a predominately psychodynamic conception of the causes of depression. Earlier, when very little else was known about the etiology of the disease, it might have been helpful to describe the action of the available drugs to patients as “correcting an imbalance” in brain neurotransmitters. Now, with so much more known or strongly suggested about the biology of depression, it is irresponsible for anyone in the medical community to describe it as simply a “chemical imbalance.” The implications of the heritability of a predisposition, the role of early life trauma, the involvement of stress hormones, the observations of neuronal atrophy, and the possible link to hippocampal neurogenesis all have implications for treatment that lead in many directions other than simply a pharmaceutical approach. These include nutrition, exercise, and certain forms of psychotherapy, especially cognitive-behavioral therapy.

But how would a depressed person know about all these treatment options? If lucky, the person might be induced, perhaps by DTC advertising, to visit a doctor, who would then describe current thinking about depression and try to determine the best treatment for the patient's situation. But doctors are extremely overworked these days, and they, too, are targeted by the advertising of pharmaceutical companies. Indeed, “medical education” is a huge industry through which these firms, at one remove, attempt to influence medical practice by the sponsoring of meetings, symposia, and journal supplements and by the well-remunerated enlistment of academic “opinion leaders” to present and to be credited as authors for what are usually ghost-written pieces. The result is a knowledge environment that is overwhelmingly dominated by the perspective of commercial interests.

The solution to this problem, of course, is education, both of physicians and of patients. A nice way to achieve this, and to refrain from infringing on anyone's free speech, would be to require that any expenditure on advertising by pharmaceutical or medical device manufacturers be matched by contributions to an unbiased education fund administered by the FDA, the National Institutes of Health (NIH), or both. This fund could be used to produce public service announcements of clear and balanced information regarding the particular therapeutic area that the company's product addresses. However it is funded, the federal government is the only entity with the resources and reach to be an effective counterweight to the commercial medical perspective. There are few roles that may be as important for a national government than the protection of its citizens' health through effective regulation of the presentation of medical information.

## References

[pmed-0030118-b1] Lacasse JR, Leo J (2005). Serotonin and depression: A disconnect between the advertisements and the scientific literature. PLoS Med.

